# Necropsy of M. Gambetta

**Published:** 1883-03

**Authors:** 


NECROPSY OF M. GAMBETTA.

According to information forwarded to us from Paris, the necropsy performed on the body of the late M. Gambetta completely justified the diagnosis of his medical attendants, and the expectant treatment which they pursued. The wounds in the hand and forearm were perfectly cicatrised, and are considered to have played no part in the cause of M. Gambetta's death. The termination of the ileum was found so much contracted, that the finger could hardly be passed into it. Bands of adhesion bound down the vermiform appendix, and there were traces of old inflammation in the cellular tissue around the cæcum. Along the course of the ascending colon the cellular tissue was infiltrated with pus, which nowhere formed a distinct abscess. In the substance of the abdominal wall, immediately adjacent to the ascending colon, but not communicating with the collection of pus around that portion of intestine, where sloughs of connective tissue, and also purulent infiltration around them, but no true abscess. There was no visible disease of the mucous membrane of the intestines. A "certain quantity of, but very little," purulent fluid was found lying free in the peritoneum, due, it was considered, to local extension of inflammation from the region of the cæcum and colon. This explains the symptoms observed during the last few days; lowering of the previously high temperature to 98.6°; pulse, 130, flatulence, hiccough, and coldness of the limbs. Intelligence was retained up to the last moment, and M. Gambetta never realized that his life was in danger. Our informant remarks that the bulletins relating to

M. Gambetta's illness have been much criticised here as being too sanguine in their tenor, but M. Gambetta, up to the last day of his life, read, or had read to him, all the newspapers, and it was therefore necessary to give somewhat vague information to the public in regard to his real condition. For many years, M. Gambetta suffered from symptoms of chronic perityphilitis extending along the ascending colon (hcnce the confusing term, "pericolitis," which has appeared in the daily papers, and become all the more confusing, through being sometimes mis-spelt pericholitis, as though the bile-duct were involved). This caused him much pain in the right flank and iliac fossa. Under the influence of the constitutional disturbance produced by the pistol-wound in the hand, but in no way through direct traumatic or pyæmic inflammation, the old-standing inflammation around the cæcum and ascending colon became acute, and assumed the form of diffuse phlegmonous inflammation ; and the abdominal walls also became the seat of the same kind of inflammation. The extension of inflammatiion to the peritoneum around the affected intestine completed the final result. It is perfectly evident, from the facts revealed by the necropsy, that surgical interference would but have hastened the fatal termination. All the remaining viscera were examined, notwithstanding their advanced stage of decomposition, hardly checked by the injection of some preservative fluid on the previous day. The lungs and heart were perfectly healthy. The liver was fatty, but not very large; there were no metastatic abscesses in any part of the body; the brain and its meninges were normal; there was no atheroma of the arteries, and only a small calcareous patch in the arch of the aorta, above the semilunar valves. The authorities present were Professors Paul Bert, Brouardel, Charcot, Cornil, Trélat, Verneuil; Doctors Lannelongue, Siredey, Fieuzal, Lionville, Mathias-Duval, Laborde, Guerdat, Gille, and M. Paul Gibier, house-surgeon. After the necropsy, performed by MM. Brouardel and Cornil, the brain of M. Gambetta was removed, in order that it might be weighed and preserved, under the directions of Dr. Charcot. This brain will ultimately be deposited in Dr. Broca's Museum of Comparative Pathology. The operation of embalming the corpse proved to be very difficult, on account of the great corpulence of the subject, and the troublesome dissection necessary before the carotids could be exposed for the introduction of the nozzle of the injecting-syringe.—*British Medical Journal.*

THE brain of Gambetta weighed 1,160 grammes. This is less than that of any person who has attained distinction since the practice of measuring the cranium and weighing its contents was inaugurated. His skull was scarcely larger than that of the people of the stone age, whose remains are found in caves in various parts of France.

